# Effects of IGF-1 on I_K_ and I_K1_ Channels via PI3K/Akt Signaling in Neonatal Cardiac Myocytes

**DOI:** 10.1155/2012/712153

**Published:** 2012-06-18

**Authors:** Richard M. Millis, Zikiar V. Alvin, Aiqiu Zhao, Georges E. Haddad

**Affiliations:** Department of Physiology & Biophysics, College of Medicine, Howard University, Washington, DC 20059, USA

## Abstract

Previous studies suggest that sarcolemmal potassium currents play important roles in cardiac hypertrophy. IGF-1 contributes to cardiac hypertrophy via activation of PI3K/Akt signaling. However, the relationships between IGF-1, PI3K/Akt signaling and sarcolemmal potassium currents remain unknown. Therefore, we tested the hypothesis that IGF-1 and PI3K/Akt signaling, independently, decrease sarcolemmal potassium currents in cardiac myocytes of neonatal rats. We compared the delayed outward rectifier (I_K_) and the inward rectifier (I_K_) current densities resulting from IGF-1 treatments to those resulting from simulation of PI3K/Akt signaling using adenoviral (Ad) BD110 and wild-type Akt and to those resulting from inhibition of PI3K signaling by LY294002. Ad.BD110 and Ad.Akt decreased I_K_ and these decrements were attenuated by LY 294002. The IGF-1 treatments decreased both I_K_ and I_K1_ but only the I_K_ decrement was attenuated by LY294002. These findings demonstrate that IGF-1 may contribute to cardiac hypertrophy by PI3K/Akt signal transduction mechanisms in neonatal rat cardiomyocytes. Failure of LY294002 to effectively antagonize IGF-1 induced decrements in I_K1_ suggests that a signal pathway adjunct to PI3K/Akt contributes to IGF-1 protection against arrhythmogenesis in these myocytes. Our findings imply that sarcolemmal outward and inward rectifier potassium channels are substrates for IGF-1/PI3K/Akt signal transduction molecules.

## 1. Introduction 

An intricate interconnected network of intracellular signaling molecules participate in regulating the electrical activity, size, and contractility of cardiac myocytes [1, 2]. Dysregulation of sarcolemmal potassium currents is known to contribute to ventricular hypertrophy, arrhythmogenesis, heart failure, and sudden death in adults [3]. Insulin-like growth factor-1 (IGF-1) is an antiarrhythmogenic and antiapoptotic modulator of cardiac myocyte hypertrophy [4]. IGF-1 is thought to contribute to cardiac myocyte hypertrophy and, at the same time protect cardiac myocytes from arrhythmogenesis and apoptosis by activating the phosphatidyl inositol-3 kinase (PI3K/Akt) cell survival intracellular signaling [4, 5]. However, the role of IGF-1 and PI3K/Akt signaling in mediating the sarcolemmal potassium current density remains unknown. The present study was, therefore, designed to test the hypothesis that IGF-1 and PI3K/Akt signaling independently modulate sarcolemmal I_K_ and I_K1_ in cardiac myocytes. 

## 2. Materials and Methods 

### 2.1. Animal Preparation

Conformity statement: as discussed in the following respective sections, all the procedures conform to the Guide for the Care and Use of Laboratory Animals published by the US National Institutes of Health (NIH) publication number 85–23, revised 1996. Pregnant female Sprague-Dawley rats of 200–250 g body weight were purchased from Charles Rivers (MA). The rats were allowed to recover and acquaint with their new environment upon arrival to the animal house of the Howard University College of Medicine for 3 days pre-partum. The pups were kept with the dams under secure, clean and controlled room temperature (70°F–74°F) with a 6:00 h to 18:00 h light cycle and the mothers were fed food and water *ad libitum*.

### 2.2. Recombinant Adenoviral Vectors

Recombinant adenoviral vectors were prepared as previously described [6, 7] by co-transfection of a shuttle vector carrying the cDNA of interest with a source of viral backbone DNA (either pAd.Track and pAd.EASY1). 

### 2.3. Preparation of Neonatal Rat Cardiac Myocytes

Neonatal cardiomyocytes preparation followed the Neonatal Cardiomyocyte Isolation System Kit (Worthington Biochemical Corporation). Freshly isolated cells were transferred to a water-jacketed CO_2_ incubator for at least 24 h before use. Cells were placed in a perfusion bath on the stage of an inverted microscope and superfused with the appropriate recording solution at a rate of 1-2 mL/min. Isolated cultured cells were incubated with IGF-1 or the LY294002 inhibitor of PI3K signaling, with normal culture media serving as the control. Cells were also transfected with adenovirus carrying the constitutive BD110 activator of PI3K or carrying the wild-type Akt mutant before use, with adenoviral enhanced green fluorescent protein (Ad.EGFP) serving as the control. 

### 2.4. Electrophysiological Effects on  I_K_  and  I_K1_

Ventricular myocytes were placed in a culture dish with an extracellular buffer containing (mM): 5 KCl, 1 MgCl2, 140 NaCl, 10 HEPES, 10 D-glucose, 1 CaCl2, 0.2 CdCl2, to which we added 0.1 *μ*M IGF-1 [8] and/or 3 *μ*M LY 294002 [8], Ad.Akt 10^11^ pfu/mL (10, 11] and/or Ad.BD110 10^11^ pfu/mL [6, 7], and pH at 7.4. The voltage dependency of I_K_ activation was studied by obtaining data for the respective current-voltage (*I*-*V*) relationships. To that end, 350 ms step voltages in 10 mV increments between −40 mV and +30 mV were applied from a holding potential of −80 mV, but with a 200 ms prepulse to −40 mV. For I_K1_, 350 ms step voltages in 10 mV increments between −140 mV and −90 mV were applied from a holding potential of −80 mV. Steady-state currents, measured at the end of each current response, were plotted as a function of the command potential. The actions of IGF-1, in the presence and absence of the constitutive Ad. BD110 or wild-type Ad.Akt activators and in the presence and absence of the LY294002 inhibitor of PI3K signaling, were analyzed for their effects on the current-voltage (*I*-*V*) relationship. LY 294002, an inhibitor of PI3K, was purchased from Cell Signaling Technology (MA). Sarcolemmal potassium currents (I_K_ and I_K1_) were expressed in current density, pA/pF. Ad.EGFP data served as the control for evaluating the effects of transfections. 

### 2.5. Statistical Analysis

The effects of treatments of neonatal cardiomyocytes with IGF-1 alone and in combination with the constitutive Ad.BD110 or wild-type Ad.Akt active vectors or with the LY294002 inhibitor of PI3K signaling on the pA/pF values at each membrane potential were compared by repeated measures ANOVA with significance set at *P* < 0.05. 

## 3. Results 

### 3.1. Effects of IGF-1 on  I_K_

**Figure 1:**
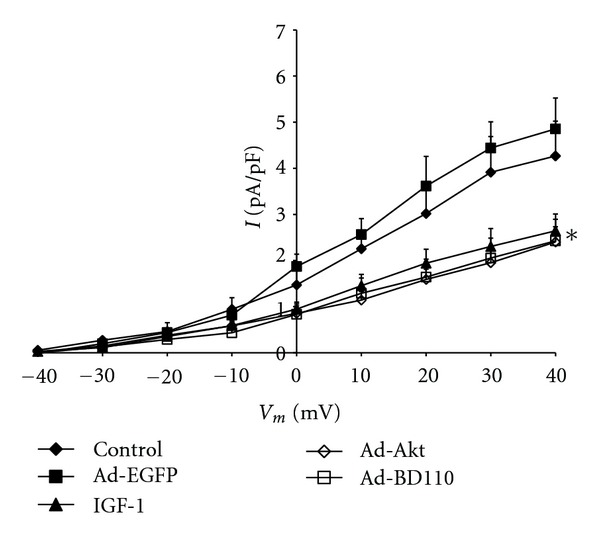
Effects of IGF-1, Ad.Akt and Ad.BD110 treatments on the I_K_-voltage relationship for normal neonatal rat cardiomyocytes. Data are presented as average current density (pA/pF) ± standard error. *Significant difference at *P* < 0.05 for control and Ad.EGFP versus IGF-1, Ad.Akt, and Ad.BD110 treatments.

**Figure 2:**
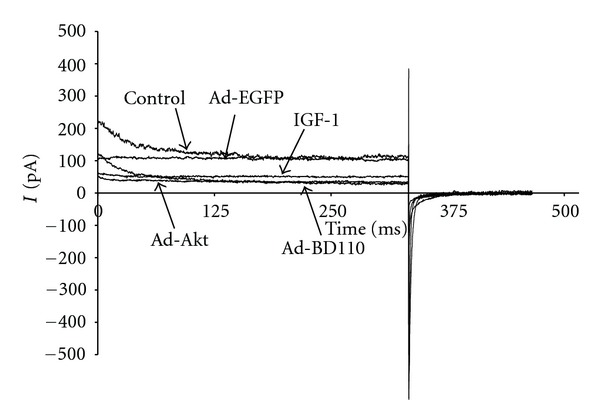
Recording representative of the time courses for the effects of IGF-1, Ad.Akt, and Ad.BD110 treatments on I_K_ of normal neonatal rat cardiomyocytes at the clamp voltage of +30 mV.

**Figure 3:**
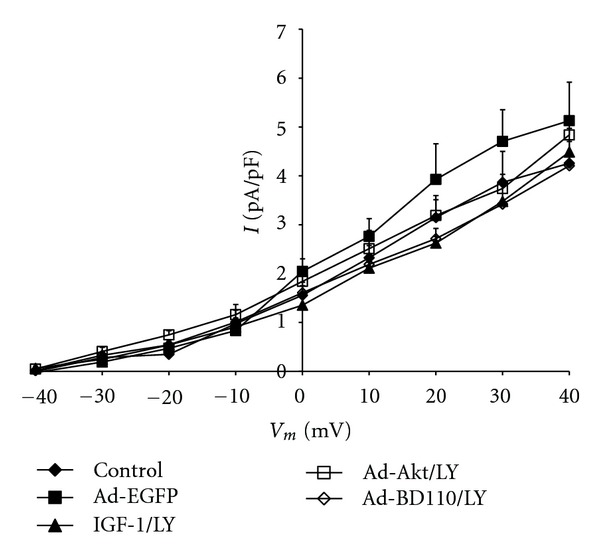
Effects of IGF-1, Ad.Akt, and Ad.BD110 in combination with LY294002 treatments on the I_K_-voltage relationship for normal neonatal rat cardiomyocytes. Data are presented as average current density (pA/pF) ± standard error.

**Figure 4:**
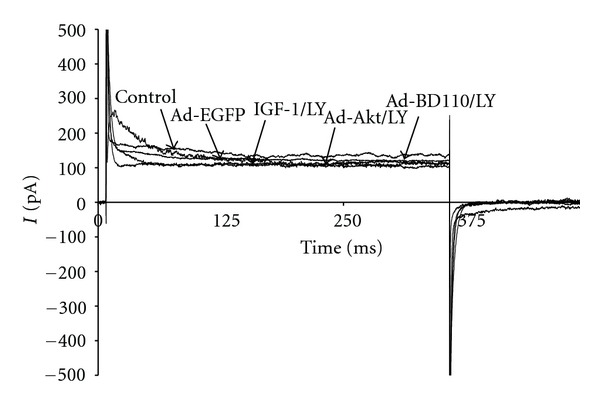
Recording representative of the time courses for the effects of IGF-1, Ad.Akt, and Ad.BD110 in combination with LY294002 treatments on I_K_ of normal neonatal rat cardiomyocytes at the clamp voltage of +30 mV. Current expressed as pA.

To evaluate the effects of IGF-1/PI3K/Akt signaling on I_K_ activity, neonatal cardiomyocytes were pharmacologically treated with the specific PI3K signaling inhibitor LY294002 or transfected for at least 24 h with adenovirus carrying the constitutive PI3K activator Ad.BD110 or the wild-type Akt activator Ad.Akt.  Figure 1 shows that treatment of cultured neonatal cardiomyocytes with IGF-1 (2.6 ± 0.4 pA/pF, 39.53 ± 2%; *n* = 8, *P* < 0.05), transfection with Ad.BD110 (2.5 ± 0.5 pA/pF, 47.91 ± 1.5%; *n* = 7, *P* < 0.05) or transfection with Ad.Akt (2.4 ± 0.3 pA/pF, 50.00 ± 1.4%; *n* = 8, *P* < 0.05) decreased I_K_ density by a similar magnitude when compared to the normal (4.3 ± 0.8 pA/pF; *n* = 10) and the Ad.EGFP (4.8 ± 0.7 pA/pF; *n* = 8) control values. Recordings representative of the current responses are shown in Figure 2.  Figure 3 also demonstrates that application of the PI3K inhibitor LY294002 attenuated the IGF-1, Ad.Akt, and Ad.BD110 effects significantly (4.5 ± 0.5 pA/pF; 4.8 ± 0.4 pA/pF; 4.2 ± 0.2 pA/pF, respectively, *P* < 0.05). Recordings representative of the current responses for the LY294002 combination treatments are shown in Figure 4. 

### 3.2. Effects of IGF-1 on  I_K1_

**Figure 5:**
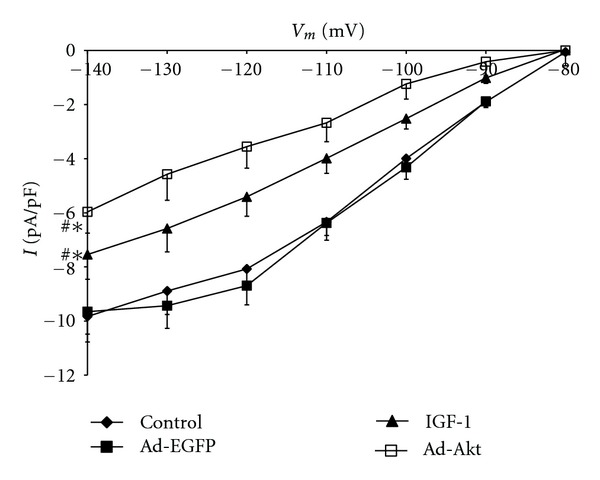
Effects of IGF-1 and Ad.Akt treatments on the I_K1_-voltage relationship for normal neonatal rat cardiomyocytes. Data are presented as average current density (pA/pF) ± standard error. ^∗#^Signficant differences at *P* < 0.05 for control and Ad.EGFP versus IGF-1 and Ad.Akt treatments.

**Figure 6:**
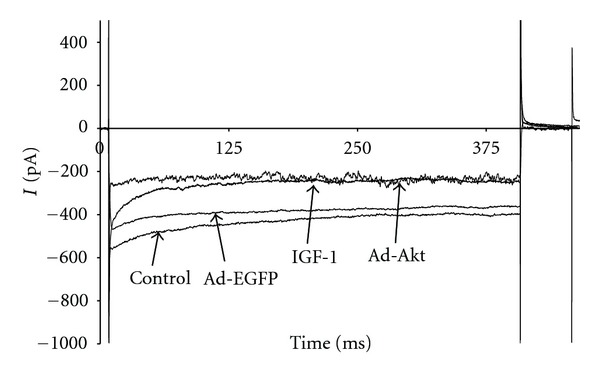
Recording representative of the time courses for the effects of IGF-1 and Ad.Akt treatments in I_K1_ of normal neonatal rat cardiomyocytes at the clamp voltage of −120 mV.

**Figure 7:**
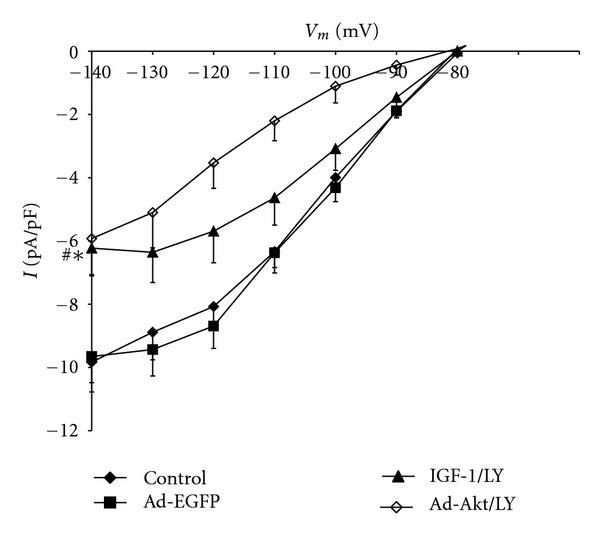
Effects of IGF-1 and Ad.Akt in combination with LY294002 treatments on the I_K1_-voltage relationship for normal neonatal rat cardiomyocytes. Data are presented as average current density (pA/pF) ± standard error. ^∗#^Significant differences at *P* < 0.05 for control and Ad.EGFP versus IGF-1 and Ad.Akt in combination with LY294002 treatments.

**Figure 8:**
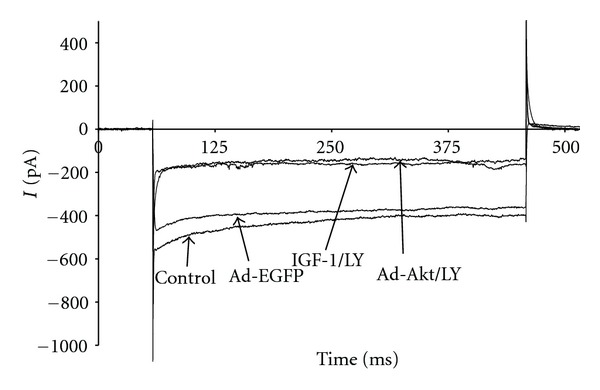
Recording representative of the time courses for the effects of IGF-1 and Ad.Akt in combination with LY294002 treatments on I_K1_ of normal neonatal rat cardiomyocytes at the clamp voltage of −120 mV. Current expressed as pA.

To determine whether the electrophysiological activity of I_K1_ is regulated by IGF-1/PI3K/Akt pathway, the cardiomyocytes were treated with IGF-1 or transfected with Ad.Akt.  Figure 5 shows that I_K1_ current density was decreased after treatments with IGF-1 by 23.46 ± 3.8% (−7.5 ± 0.9 pA/pF; *n* = 8, *P* < 0.05) or, after transfection with Ad.Akt, by 38.54 ± 1.8% (−5.9 ± 0.9 pA/pF; *n* = 6, *P* < 0.05), compared to the normal control (−9.8 ± 0.9 pA/pF; *n* = 10) and to the Ad.EGFP control values (−9.7 ± 0.8 pA/pF; *n* = 8). Recordings representative of the current responses are shown in Figure 6.  Figure 7 shows that application of the PI3K inhibitor LY294002 failed to alter both the IGF-1 and the Ad.Akt effects on I_K1_ density. Recordings representative of the current responses for the LY294002 combination treatments are shown in Figure 8.

## 4. Discussion 

During ventricular hypertrophy, changes in ion channel activity are mediated by intracellular signal transduction mechanisms connected to the addition or subtraction of the fetal gene program [9]. IGF-1 is known to activate PI3K and its downstream target Akt, a serine threonine kinase, also known as protein kinase [10–12]. IGF-1 binding to its receptor with downstream activation of PI3K and Akt has been demonstrated [13, 14]. IGF-1 is known to play a major role in cellular proliferation and differentiation [15–17]. Treatment of cultured newborn rat cardiac myocytes with IGF-1 is reported to activate specific genes that accompany electrophysiological changes [18] and IGF-1 mRNA and protein to increase in parallel with alterations in sarcolemmal ion currents [19]. Furthermore, overexpression of Akt appears to induce changes in the electrophysiological properties of the hearts of transgenic mice [20, 21]. These findings suggest important roles for IGF-1, as well as significant gaps in knowledge concerning IGF-1 regulation of sarcolemmal potassium channels by the PI3K/Akt cell survival signal transduction pathway. 

In the present study, we characterized the alterations of potassium channel current density (I_K_ and I_K1_) associated with treatments of cultured neonatal rat cardiomyocytes with IGF-1 alone and in combination with two activators (Ad.BD110, Ad.Akt) and one inhibitor of the PI3K/Akt signaling pathway. We demonstrated significant decrements in I_K_ and I_K1_ current density by IGF-1. The effects of IGF-1 on I_K_ seemed to be mediated, largely, by PI3K/Akt signaling. Although I_K1_ was found to be sensitive to treatment with IGF-1 and Ad.Akt, LY294002 had no significant effect on the IGF-1-induced decrement in I_K1_ density. This finding suggests that IGF-1 might modulate I_K1_ by another mechanism cross-talking downstream from PI3K/Akt signaling and that the inward rectifier channel (Kir) may not be a direct substrate for the PI3K/Akt signaling molecules. 

The stimulators of intracellular signal transduction molecules Ad.BD110, a constitutively active PI3K and Ad.Akt, wild-type Akt, decreased I_K_ density. The wild-type Ad.Akt also decreased I_K1_ density. The Akt simulator Ad.Akt also decreased I_K1_ density. However, the PI3K inhibitor LY294002 effectively antagonized the effects of IGF-1, Ad.BD110, and Ad.Akt on I_K_ density but failed to antagonize the effects of IGF-1 and Ad.Akt on I_K1_ density. A limitation of this study is that the effects of Ad.BD110 on I_K1_ density were not determined. Nevertheless, these results imply that both PI3K and Akt signaling pathway molecules play roles in the decrement in I_K_ density associated with IGF-1, an effect known to increase the duration of cardiac myocyte action potentials. However, although Akt signaling seems to play a role in the decrement in I_K1_ density associated with IGF-1, this effect appears to be independent of PI3K activity.

The IGF-1/PI3K/Akt signaling pathway is the best characterized example of intracellular signal transduction contributing to physiological and pathological cardiac hypertrophies. IGF-1/PI3K/Akt signaling molecules are reported to be critical for exercise-induced cardiac hypertrophy [5, 13], in concert with AMP-activated kinase [22]. The effects of IGF-1/PI3K/Akt signaling on I_K_ and I_K1_ in exercise-hypertrophied cardiomyocytes have not been demonstrated. It is tempting to speculate that IGF-1/PI3K/Akt signaling mediates physiological growth and hypertrophy and antagonizes pathological hypertrophy [13]. However, the mechanisms underlying pathological hypertrophy appear to be more complex. We have demonstrated that IGF-1/PI3K/Akt signaling also plays a role in eccentrically hypertrophied adult rat cardiomyocytes, decreasing the current densities of both I_K_ and I_K1_ [8, 23]. IGF-1 appears to upregulate the transient outward potassium current associated with the early repolarization of phase 1 of the ventricular myocyte action potential in normal neonatal rat cardiomyocytes [24]. Although Ito initiates repolarization, the delay in completing repolarization and prolonging depolarization is mainly a function of the phase 3 outwardly rectifying current, I_K_. We previously reported that downregulation of ATP-sensitive potassium channel current (I_KATP_) is a characteristic of volume-overloaded, eccentrically hypertrophied adult rat cardiac myocytes which is thought to increase the duration of ventricular action potentials and increase inotropy of the myocytes [25]. The IGF-1 induced decrement in I_K_ described in the present study might also contribute to prolonging ventricular action potentials. This IGF-1 induced decrement in I_K_ was simulated by activating the PI3K/Akt pathway using both Ad.BD110 and Ad.Akt and has been shown to be sensitive to inhibition by the PI3K inhibitor LY294002. These findings suggest that PI3K/Akt signaling molecules could serve as substrates for inhibiting cardiomyocyte hypertrophy in neonatal cardiac myocytes. I_K1_ is the inwardly rectifying potassium current responsible for restoring the resting membrane potential and excitability of ventricular myocytes. Upregulation of I_K1_ is reported to be a significant contributor to the cardiac arrhythmias associated with hypertrophy of cardiac myocytes [26, 27]. The IGF-1-induced decrement in I_K1_, simulated by activating the PI3K/Akt signaling pathway using Ad.Akt and insensitive to the PI3K inhibitor LY294002, is, therefore, likely to be dependent on the signaling molecules associated with an alternate or adjunctive signaling pathway such as AMP-activated kinase shown to play a role in cardiac myocyte hypertrophy [22]. The IGF-1-induced decrement in I_K1_, by virtue of the role of I_K1_ on resting membrane excitability, is likely to protect against the arrhythmogenesis associated with cardiac myocyte hypertrophy [5]. Decreasing the inward potassium resting potential stabilization current may, therefore, increase the threshold for ventricular arrhythmogenesis, and the Kir channels in neonatal cardiac myocytes might, therefore, function as substrates for treatments to inhibit the predictable, potentially fatal ventricular arrhythmias associated with adult cardiac hypertrophy [28–31] and with hypertrophy associated with neonatal cardiomyopathy [32]. 

We did not look for or observe any significant changes in the size of the neonatal cardiac myocytes that we studied. These normal neonatal cardiac myocytes were used as a model to determine whether the decrements in I_K_ and I_K1_ density, which we previously observed in eccentrically hypertrophied adult rat cardiac myocytes and not in normal adult rat cardiac myocytes exposed to IGF-1 [8, 23] were effects inherent or specific to such hypertrophied cardiac myocytes. The results of the present study, demonstrating decreased I_K_ and I_K1_ density in normal neonatal cardiac myocytes, suggest that similar effects could be elicited from not only eccentrically hypertophied adult rat cardiomyocytes, but also from normal neonatal rat cardiomyocytes treated with, or exposed to, IGF-1.

## 5. Conclusions 

The present study demonstrates that stimulation of the IGF-1/PI3K/Akt cell survival signaling pathway decreases activities of the sarcolemmal I_K_ outward and the I_K1_ inward rectifier currents of neonatal cardiac myocytes. These results in normal neonatal rat cardiac myocytes are in agreement with our previous reports in volume-overloaded, eccentrically hypertrophied adult rat cardiac myocytes. Our findings imply that sarcolemmal outward and inward rectifier potassium channels are substrates for IGF-1/PI3K/Akt signal transduction molecules. Comparisons of the effects of cellular signal transduction molecules on normal and hypertrophied cardiomyocytes may be useful experimental models for elucidating the sarcolemmal ion channel substrates and regulatory mechanisms underlying both neonatal and adult cardiac cardiomyopathies.

## References

[1] Frey N, Olson EN (2003). Cardiac hypertrophy: the good, the bad, and the ugly. *Annual Review of Physiology*.

[2] Hunter JJ, Chien KR (1999). Signaling pathways for cardiac hypertrophy and failure. *New England Journal of Medicine*.

[3] Nabauer M, Kaab S (1998 ). Potassium channel down-regulation in heart failure. *Cardiovascular Research*.

[4] Van Empel VPM, De Windt LJ (2004). Myocyte hypertrophy and apoptosis: a balancing act. *Cardiovascular Research*.

[5] Ellison GM, Waring CD, Vicinanza C, Torella D (2012). Physiological cardiac remodelling in response to endurance exercise training: cellular and molecular mechanisms. *Heart*.

[6] Hajjar RJ, Kang JX, Gwathmey JK, Rosenzweig A (1997). Physiological effects of adenoviral gene transfer of sarcoplasmic reticulum calcium ATPase in isolated rat myocytes. *Circulation*.

[7] Hajjar RJ, Schmidt U, Kang JX, Matsui T, Rosenzweig A (1997). Adenoviral gene transfer of phospholamban in isolated rat cardiomyocyte: rescue effects by concomitant gene transfer of sarcoplasmic reticulum Ca^2+^- AtPase. *Circulation Research*.

[8] Teos LY, Zhao A, Alvin Z, Laurence GG, Li C, Haddad GE (2008). Basal and IGF-I-dependent regulation of potassium channels by MAP kinases and PI3-kinase during eccentric cardiac hypertrophy. *American Journal of Physiology, Heart and Circulatory Physiology*.

[9] McMullen JR, Jennings GL (2007). Differences between pathological and physiological cardiac hypertrophy: novel therapeutic strategies to treat heart failure. *Clinical and Experimental Pharmacology and Physiology*.

[10] Jia G, Aggarwal A, Yohannes A, Gangahar DM, Agrawal DK (2011). Cross-talk between angiotensin II and IGF-1-induced connexin 43 expression in human saphenous vein smooth muscle cells. *Journal of Cellular and Molecular Medicine*.

[11] Lee WJ (2009). Insulin-like growth factor-I-induced androgen receptor activation is mediated by the PI3K/Akt pathway in C2C12 skeletal muscle cells. *Molecules and Cells*.

[12] Romanelli RJ, Mahajan KR, Fulmer CG, Wood TL (2009). Insulin-like growth factor-I-stimulated Akt phosphorylation and oligodendrocyte progenitor cell survival require cholesterol-enriched membranes. *Journal of Neuroscience Research*.

[13] DeBosch B, Treskov I, Lupu TS (2006). Akt1 is required for physiological cardiac growth. *Circulation*.

[14] McMullen JR, Shioi T, Huang WY (2004). The Insulin-like growth factor 1 receptor induces physiological heart growth via the phosphoinositide 3-kinase(p110*α*) pathway. *Journal of Biological Chemistry*.

[15] Hanson MC, Fath KA, Alexander RW, Delafontaine P (1993). Induction of cardiac insulin-like growth factor I gene expression in pressure overload hypertrophy. *American Journal of the Medical Sciences*.

[16] Huang CY, Hao LY, Buetow DE (2002). Insulin-like growth factor-II induces hypertrophy of adult cardiomyocytes via two alternative pathways. *Cell Biology International*.

[17] Isgaard J, Wahlander H, Adams MA, Friberg P (1994). Increased expression of growth hormone receptor mRNA and insulin-like growth factor-I mRNA in volume-overloaded hearts. *Hypertension*.

[18] Ito H, Hiroe M, Hirata Y (1993). Insulinlike growth factor-I induces hypertrophy with enhanced expression of muscle specific genes in cultured rat cardiomyocytes. *Circulation*.

[19] Donohue TJ, Dworkin LD, Lango MN (1994). Induction of myocardial insulin-like growth factor-I gene expression in left ventricular hypertrophy. *Circulation*.

[20] Matsui T, Li N, Wu JC (2002). Phenotypic spectrum caused by transgenic overexpression of activated Akt in the heart. *Journal of Biological Chemistry*.

[21] Shioi T, McMullen JR, Kang PM (2002). Akt/protein kinase B promotes organ growth in transgenic mice. *Molecular and Cellular Biology*.

[22] Kim J, Wende AR, Sena S (2008). Insulin-like growth factor I receptor signaling is required for exercise-induced cardiac hypertrophy. *Molecular Endocrinology*.

[23] Zhao A, Alvin Z, Laurence G, Li C, Haddad GE (2010). Cross-talk between MAPKs and PI-3K pathways alters the functional density of I_K_ channels in hypertrophied hearts. *Ethnicity & Disease*.

[24] Guo W, Kada K, Kamiya K, Toyama J (1997). IGF-I regulates K+-channel expression of cultured neonatal rat ventricular myocytes. *American Journal of Physiology, Heart and Circulatory Physiology*.

[25] Alvin ZV, Millis RM, Hajj-Mousssa W, Haddad GE (2011). ATP-sensitive potassium channel currents in eccentrically hypertrophied cardiac myocytes of volume-overloaded rats. *International Journal of Cell Biology*.

[26] Dhamoon AS, Jalife J (2005). The inward rectifier current (I_K1_) controls cardiac excitability and is involved in arrhythmogenesis. *Heart Rhythm*.

[27] Li J, McLerie M, Lopatin AN (2004). Transgenic upregulation of I_K1_ in the mouse heart leads to multiple abnormalities of cardiac excitability. *American Journal of Physiology, Heart and Circulatory Physiology*.

[28] Bignolais O, Quang KL, Naud P (2011 ). Early ion-channel remodeling and arrhythmias precede hypertrophy in a mouse model of complete atrioventricular block. *Journal of Molecular and Cellular Cardiology*.

[29] Lynch JJ, Sanguinetti MC, Kimura S, Bassett AL (1992). Therapeutic potential of modulating potassium currents in the diseased myocardium. *FASEB Journal*.

[30] Panyasing Y, Kijtawornrat A, del Rio C, Carnes C, Hamlin RL (2010). Uni- or bi-ventricular hypertrophy and susceptibility to drug-induced torsades de pointes. *Journal of Pharmacological and Toxicological Methods*.

[31] Yang KC, Jay PY, McMullen JR, Nerbonne JM (2012 ). Enhanced cardiac PI3Kalpha signalling mitigates arrhythmogenic electrical remodelling in pathological hypertrophy and heart failure. *Cardiovascular Research*.

[32] Chang KTE, Taylor GP, Meschino WS, Kantor PF, Cutz E (2010). Mitogenic cardiomyopathy. A lethal neonatal familial dilated cardiomyopathy characterized by myocyte hyperplasia and proliferation. *Human Pathology*.

